# Targeting the Activin Receptor Signaling to Counteract the Multi-Systemic Complications of Cancer and Its Treatments

**DOI:** 10.3390/cells10030516

**Published:** 2021-02-28

**Authors:** Juha J. Hulmi, Tuuli A. Nissinen, Fabio Penna, Andrea Bonetto

**Affiliations:** 1 Faculty of Sport and Health Sciences, NeuroMuscular Research Center, University of Jyväskylä, 40014 Jyväskylä, Finland; tuuli.nissinen@utu.fi; 2 Department of Clinical and Biological Sciences, University of Turin, 10125 Turin, Italy; fabio.penna@unito.it; 3 Department of Surgery, Indiana University School of Medicine, Indianapolis, IN 46202, USA

**Keywords:** cancer cachexia, tumor, chemotherapy, myostatin, activins, muscle wasting, survival, mortality, multi-organ

## Abstract

Muscle wasting, i.e., cachexia, frequently occurs in cancer and associates with poor prognosis and increased morbidity and mortality. Anticancer treatments have also been shown to contribute to sustainment or exacerbation of cachexia, thus affecting quality of life and overall survival in cancer patients. Pre-clinical studies have shown that blocking activin receptor type 2 (ACVR2) or its ligands and their downstream signaling can preserve muscle mass in rodents bearing experimental cancers, as well as in chemotherapy-treated animals. In tumor-bearing mice, the prevention of skeletal and respiratory muscle wasting was also associated with improved survival. However, the definitive proof that improved survival directly results from muscle preservation following blockade of ACVR2 signaling is still lacking, especially considering that concurrent beneficial effects in organs other than skeletal muscle have also been described in the presence of cancer or following chemotherapy treatments paired with counteraction of ACVR2 signaling. Hence, here, we aim to provide an up-to-date literature review on the multifaceted anti-cachectic effects of ACVR2 blockade in preclinical models of cancer, as well as in combination with anticancer treatments.

## 1. Introduction

Skeletal muscle is essential for locomotion, breathing, maintenance of bone mass and strength, and it plays a central role in whole body metabolism, acting as a target for glucose disposal and serving as an amino acid reservoir [1,2]. Muscle size, quality and function have been strongly related to risk of mortality and overall outcomes in different diseases and wasting conditions [3,4,5]. Regardless, the role of skeletal muscle tissue remains underappreciated in health and disease [2].

Wasting syndrome associated with disease states, such as cancer, is referred to as cachexia [6]. Cachexia is a multifactorial syndrome characterized by loss of body mass due to wasting of muscle, often also accompanied by loss of adipose tissue and increased inflammation [7]. Cancer cachexia induces substantial alterations in many tissues, organs and metabolic pathways [6]. Many of these alterations are compensatory adaptations aimed to restore the tissue homeostasis disrupted by tumor and antitumor treatments; however, in certain conditions, such derangements ultimately become harmful to the patient, resulting in energetic inefficiency and wasting [6].

Cancer cachexia is associated with poor prognosis and increased chemotherapy toxicity, while the latter can further aggravate muscle wasting and thus potentially compromise cancer therapies [4]. Even though the poor prognosis associated with cachexia has been acknowledged for almost a century [8], it was only quite recently that research started to focus on the importance of skeletal muscle mass in cancer, mainly as a target to design potential therapies to counteract cachexia.

Two hallmark studies by Benny Klimek et al. [9] and Zhou et al. [10] demonstrated that blockade of activin receptor 2B (ACVR2B) ligands successfully prevented cachexia in tumor-bearing mice. Interestingly, prevention and reversal of cachexia were associated with improved survival in tumor-bearing mice without effects on tumor growth [10]. These findings highlighted the importance of maintaining muscle mass in experimental cancer cachexia and proposed blocking of ACVR2 signaling as a potential therapeutic strategy to counteract cachexia, thereby also prolonging survival. Similar pro-survival properties of blocking ACVR2B ligands or the receptor have since been validated by others [11,12,13], although together, these studies emphasized the idea that improved survival may be a multi-systemic phenomenon, and thus more complex than previously thought. Additional studies from our groups have recently shown that blocking ACVR2 ligands is also effective in preventing muscle wasting in different murine models of chemotherapy-associated cachexia [14,15,16,17]. Altogether, the translational value of these preclinical studies is also supported by evidence that blocking these ligands in healthy humans promotes larger muscles [18].

Despite all this, whether targeting ACVR2 signaling represents a putative therapeutic strategy for the preservation of muscle mass and function in cachexia remains partially unclear, especially in light of the recent unsuccesful translation of myostatin/activin inhibitors in the treatment of Duchenne muscular dystrophy and other muscle wasting conditions in humans (see References [18,19]). Here, we provide a proof of concept and an up-to-date literature review on the effects of ACVR2 counteraction in preclinical cancer- and chemotherapy-induced cachexia models with a special emphasis on the multi-tissue and multi-systemic effects possibly contributing to improved survival.

## 2. Cachexia Induced by Cancer or Chemotherapy

### 2.1. Cancer Cachexia and Skeletal Muscle Wasting

Cancer cachexia is defined as “a multifactorial syndrome characterized by an ongoing loss of skeletal muscle mass (with or without loss of fat mass) that cannot be fully reversed by conventional nutritional support and leads to progressive functional impairment” [7]. Considering the number of patients affected by such comorbidity (30–80% depending on the tumor type [20,21]), the poor prognosis related to it, and the fact that no effective therapies are currently available, cancer cachexia represents an important field of investigation. Cachexia arises from a variable combination of reduced food intake and abnormal metabolism, including systemic inflammation. The most advanced stage of cachexia, i.e., refractory cachexia, is characterized by progressive catabolism and unresponsiveness to anticancer treatments. Unfortunately, during this phase, patients are unlikely to benefit from interventions targeted at reversing muscle wasting or cachexia [7]; hence, there is a need to identify early targets for intervention.

Cachexia is associated with progressive wasting in many human cancers and pre-clinical cancer cachexia models utilizing rodents [13,21,22,23,24,25,26] and is well-known as an independent risk factor for mortality, as well as for increased chemotherapy-related toxicity [4,27,28,29]. In addition to negative effects on prognosis and tolerance to anti-cancer therapies, muscle wasting associated with cancer cachexia drastically impairs the quality of life and functional capacity of cancer patients and induces weakness, fatigue and exercise intolerance [30]. Muscle wasting in cancer cachexia has been attributed to increased protein degradation [12,30,31,32,33] and/or decreased protein synthesis [13,30,31,32,33,34,35]. A potential involvement of impaired regeneration in skeletal muscle has also been a recent subject of investigations [36]. Overall, the relative contribution of each of these mechanisms may depend on type of cancer and the stage of cachexia [31,33].

### 2.2. Chemotherapy and Skeletal Muscle Wasting

Despite the recent progress in the development of new therapies for cancer, cytotoxic chemotherapy remains the preferred treatment strategy for most tumors, irrespective of its associated toxicities. In line with this, the interaction among tumor, host and anticancer treatments is usually critical for the overall outcome. If the tumor responds to the treatment, then the applied anticancer therapy, such as chemotherapy, is likely to alleviate cachexia and to improve patient’s quality of life [4,32,37]. For instance, in tumor-bearing mice, chemotherapy with antitumor effects has been found to restore muscle protein synthesis [32]. However, in some cases, as already reported in tumor-bearing animals, the negative effects of chemotherapy can exacerbate the negative nitrogen balance despite its antitumor activity [38], thus also underlining the specificity of the effects of chemotherapeutic agents and their interaction with the tumor. However, to better clarify chemotherapy interaction with skeletal muscle mass, it is also important to evaluate its independent effects in the absence of a tumor. Indeed, it has been shown that even before tumor regression occurs, different anticancer therapies, including cytotoxic chemotherapy, surgery, radiation therapy, androgen-deprivation therapy, or targeted therapies, may cause muscle wasting and thus aggravate the cachectic phenotype [4,37,39].

Many different chemotherapeutic agents are used, alone or in combination with other agents, to treat cancer. Among these, doxorubicin, a widely used anthracycline chemotherapeutic agent, is used to treat different cancers [40]. Unfortunately, in addition to its antitumor effects, doxorubicin has deleterious effects on noncancerous tissues, with cardiotoxicity being its most well-known side effect and limiting its clinical use [40,41,42]. However, even with this limited dosage, doxorubicin accumulates into skeletal muscle [15,43], and studies in both humans [41] and animals [44,45,46,47,48,49,50,51,52,53] have usually reported muscle weakness, fatigue, dysfunction and atrophy after chemotherapy with doxorubicin alone or combined with other cytostatic agents.

High doses of doxorubicin have been shown to activate markers of proteolysis [51,54]. However, the degree of proteolysis may be small compared with other muscle wasting conditions [55,56], as typical “atrogene” expression signature was not observed in murine skeletal muscle acutely after a single dose of chemotherapy [14]. In addition, increased markers of autophagy [44,54] and apoptosis [44,51,54] have been observed in muscles of rodents treated with doxorubicin-based chemotherapy regimens.

In addition to doxorubicin, the muscle effects of other chemotherapeutics have been recently investigated in rodents. Barreto et al. [17] were the first to report that chronic administration of clinically relevant doses of Folfiri, a combination of 5-fluorouracil, leucovorin and irinotecan frequently prescribed for the treatment of solid tumors, promoted the occurrence of a cachexia-like syndrome in healthy mice, including transient loss of food intake, body and muscle weight loss, as well as muscle weakness. Interestingly, dramatic bone loss was also described in healthy animals receiving Folfiri [57]. The same group showed that among the mechanisms responsible for such muscle phenotype consequential to Folfiri administration were activation of mitogen-activated protein kinases (MAPKs) and mitochondrial abnormalities [17,58], the latter mostly responsible for perturbations in the energy metabolism [59]. Similarly, treatment with the multi-kinase inhibitors (MKIs) regorafenib and sorafenib, used as second-line treatment for solid tumors, was shown to drive muscle toxicities in in vivo conditions, including muscle wasting and weakness. Of note, cardiac defects, such as reduced left ventricular mass, internal diameter, posterior wall thickness and stroke volume, were also described [60]. Additionally, platinum-based chemotherapeutics were found to cause similar musculoskeletal deficits, with cisplatin and carboplatin triggering skeletal muscle atrophy and marked weakness, along with extensive bone resorption, which was completely abolished by combination with bone-targeting anti-resorptive bisphosphonates [61,62,63].

Similar to evidence suggesting a pro-survival role of skeletal muscle mass in cancer, many studies have proposed a role of muscle mass also in the metabolization of and the tolerance to anticancer drugs. Indeed, it was reported that sarcopenia, low muscle size, or low lean body mass associate with increased incidence of toxicity in patients receiving chemotherapy [27,28,29,64,65]. Keeping in mind that weight loss and muscle wasting have been suggested to influence the response to treatment [66,67], it appears that the toxic effects of chemotherapy in patients with low muscularity may require dose limitations, delays, or even termination of the treatments, thus obviously hindering their efficacy [7,27,28]. However, the association between lean body mass and chemotherapy toxicity has not been observed in all studies [68], and it has been speculated that the increased toxicity reported in many studies may result from conventional dosing based on body surface area rather than body composition. Based on this idea, patients with low fat-free mass in relation to body surface area, comprising especially women and obese subjects, may present lower volume of distribution of the drug, thereby often resulting in overdosing and increased toxicity [28,29,69,70]. Thus, considering both the potentially harmful effect of chemotherapy on muscle tissue and the negative effect of low muscle mass on the outcome of the treatment, it would be of great importance to find effective therapies to counteract muscle wasting that would potentially provide more time to treat the underlying disease. Moreover, preservation of muscle mass may help to reduce the toxic effects of chemotherapy and thus improve survival in cancer patients [71]. Considering that starting chemotherapy administration as early as possible after cancer diagnosis is paramount for the oncologists, for the future, we envisage that supportive oncological treatments aimed at preserving muscle mass even in the absence of overt cachexia will begin simultaneously.

Unfortunately, our current knowledge of chemotherapy-associated effects on whole body mass and metabolism is limited, therefore highlighting the need for an up-to-date comprehensive review of the muscle wasting effects of different anticancer therapies. Interestingly, Talbert et al. showed that not all cancer treatments per se induce muscle atrophy [72]. More studies are also needed as most of the studies conducted so far have investigated the effects of chemotherapy in healthy organisms, therefore highlighting the possibility that some of the side effects produced by anticancer drugs may be different in the presence of a tumor. Furthermore, in addition to the effects of the anticancer treatments on muscle tissue, the development of cachexia may cause some limitations to the anticancer therapies, thus potentially hindering the effectiveness of the treatment and the overall outcome [4,27,28,29]. These effects are reviewed in the next sections.

### 2.3. Cachexia and Survival: The Role of Skeletal Muscle Wasting

The development of cachexia is associated with impaired prognosis and survival in cancer patients [4,27,67,73]. A number of studies have found association between the loss of body mass [5,67,74,75,76] and skeletal muscle [77,78,79,80,81,82] and the overall survival, thus suggesting that the rate of wasting might play a critical role. However, while the role of cachexia seems to be rather clear, the independent association between muscle loss and survival has not been found in all studies [66], thus suggesting a more complex picture.

Many [5,27,69,83,84,85,86,87,88,89,90,91,92] but not all [80,93,94,95,96,97] studies have reported that low muscle mass or cross-sectional area at baseline independently predict poorer survival in cancer patients. Moreover, poor muscle quality has been shown to be associated with shorter survival in cancer [5,96]. However, the role of larger muscle mass as a prognostic factor is less clear, as pharmacologically mediated increase in muscle mass only before the cachectic stimulus was not sufficient to provide a survival benefit [13]. In addition to muscles involved in locomotion, usually investigated in human studies, preclinical studies have shown that cachexia also affects other vital muscle groups, such as cardiac and respiratory muscles. For instance, formation of hepatic colorectal cancer metastases was recently described concurrent to evidence of cardiac dysfunctions [98]. On the other hand, atrophy and weakness of the diaphragm [13,24,99], the major respiratory muscle, accompanied by ventilatory dysfunction [99], have been observed in a murine model of cancer cachexia. These may potentially contribute to the impaired survival associated with muscle wasting [100,101,102], although further investigations are required to conclusively validate the hypothesis that muscle mass and function play a causal role in cancer survival.

In support of a causal link between cachexia and mortality, prevention of muscle wasting has been associated with improved survival in a number of pre-clinical murine models of cancer cachexia [10,11,12,13,103,104,105,106,107,108,109]. Inhibition of nuclear factor-κB (NF-κB) signaling in muscle [104] or tumour necrosis factor (TNF)-like weak inducer of apoptosis/ fibroblast growth factor-inducible 14 (TWEAK/Fn14) signaling in the tumor [106], blockade of growth differentiation factor 15 (GDF15) [107], treatment with histone deacetylase inhibitors [109], or counteraction of myostatin and activins [10,11,12,13,105,110] have resulted in prevention of muscle wasting and improved survival in different murine models of cancer cachexia. From a speculative perspective, improved survival in the above-mentioned studies is likely due to a complex multi-organ effect that goes beyond the rescue of muscle mass. Conversely, sparing muscle mass may play an essential role in the overall health of the host, thus better coping with tumor growth.

**Figure 1:**
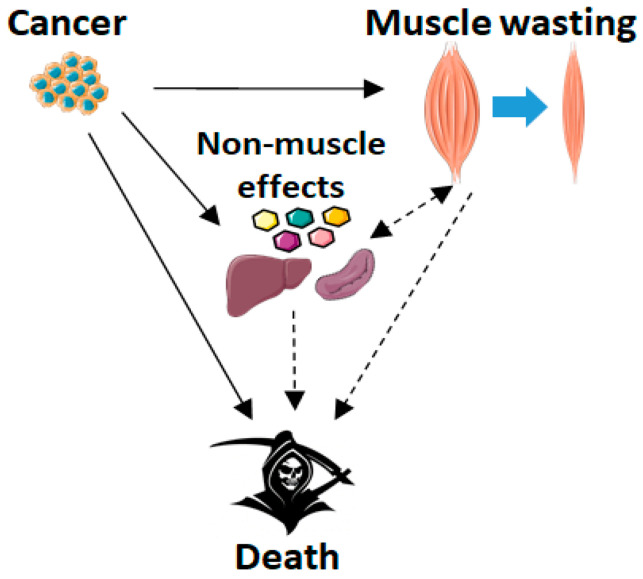
Hypothesis of causality between cancer cachexia and death. Dashed lines indicate inferred mechanisms requiring further investigation. Images were adapted from https://smart.servier.com (accessed on 23 February 2021).

In all the human and animal studies, there are also other factors affecting survival besides muscle per se. Indeed, despite the association between low muscle mass, wasting and mortality risk, it is still debated whether this link is actually causal, and there is still no consensus on the mechanisms by which cachexia causes premature death [111]. Also, cachexia never exists without the underlying disease and it is possible that it simply represents an epiphenomenon that is secondary to the disease or its risk factors, and that the disease causes death independent of cachexia [111] (see Figure 1 below).

## 3. Activin Receptor Ligands in Cancer- and Chemotherapy-Induced Cachexia

### 3.1. Discovery and Function

The Transforming Growth Factor β (TGF-β) superfamily consists of more than 30 growth factors representing TGF-βs, growth and differentiation factors (GDFs), bone morphogenetic proteins (BMPs), activins and nodal [112]. The best known activin receptor ligands, myostatin, activins and GDF11, are reviewed below.

Myostatin (also known as GDF-8) was discovered in 1997, when its expression during embryonic development and in adult muscle was first described [113]. It was found that myostatin mRNA was expressed almost exclusively in skeletal muscle tissue and that homozygous disruption of the myostatin gene resulted in individual muscles to become 2–3 times larger [113]. In addition, mutations in the myostatin gene were described concurrent to a double-muscled phenotype in cattle [114,115,116]. These results led to the conclusion that myostatin acts specifically as a negative regulator of muscle growth. Indeed, blockade of endogenous myostatin was shown to drive muscle hypertrophy [117,118,119] and increased strength also in adult muscle [118]. Conversely, overexpression of myostatin was found to cause muscle atrophy [120,121], showing that myostatin is able to regulate muscle size also in adults.

GDF11, closely related to myostatin, was found to be expressed in many tissues and to play a role in skeletal patterning and development of bone and nervous system, as well as in aging and in different disease states [122,123]. Unlike myostatin, whose effects seem to be mostly restricted to skeletal muscle, overexpression of GDF11 was also found to equally cause atrophy in skeletal and cardiac muscles [124]. Furthermore, muscle-specific deletion of the GDF11 gene was not shown to affect skeletal muscle size, indicating that at least muscle-derived endogenous GDF11 may not be vital for regulation of skeletal muscle size [125], although some controversy exists concerning the role of GDF11 in the regulation of skeletal muscle tissue during the whole lifespan [126]. Thus, the effects of GDF11 and its inhibition in adult skeletal muscle require further investigation.

Activins are pleiotropic proteins belonging to the TGF-β superfamily [127]. Among the activins, the best characterized, activin A, was discovered in 1986 by Vale and colleagues from ovarian follicular fluid [128]. Their name originates from their ability to stimulate the release of follicle-stimulating hormone from the pituitary gland, in contrast to inhibin, which, on the contrary, inhibits its release [127]. Activins play important roles in reproduction and embryonic development [127]. In addition, activins and their receptors are present in virtually all mammal body systems, and thus they have varying functions all over the body in normal physiology and in response to injury or disease [127,129]. For example, activin A has been shown to have important effects on multiple extra-reproductive systems, including the brain, cardiac, renal, digestive, immune and respiratory systems, as well as the musculoskeletal system [127]. The effects on the muscular system are reviewed in more detail below.

Mice deficient in inhibin, a competitive antagonist for activin, have been shown to develop gonadal tumors and severe cachexia. This has been found to be associated with increased levels of activin A and B, secreted from the tumors, that potentially contribute to development of cachexia [130]. Like myostatin, activin A acts as a negative regulator of muscle growth during both development and adulthood. This is supported by studies showing that overexpression of activin A leads to muscle atrophy [131,132], while heterozygous loss-of-function mutation in the activin A gene [133], as well as activin A antagonism via overexpression of activin A pro-domain [134], result in increased muscle mass. Both activin A and its receptor are expressed in adult skeletal muscle. Their effects on skeletal muscle appear to include inhibition of protein synthesis and promotion of protein breakdown, thus negatively regulating muscle size [127]. Moreover, activin A overexpression can decrease muscle contractile function and force production and increase fibrosis in skeletal muscle [131]. Similar to activin A, activin B overexpression was shown in association with muscle atrophy [131], and inhibition by overexpression of activin B pro-domain [134] resulted in muscle hypertrophy, suggesting that activin B can act as a negative regulator of muscle size.

**Figure 2:**
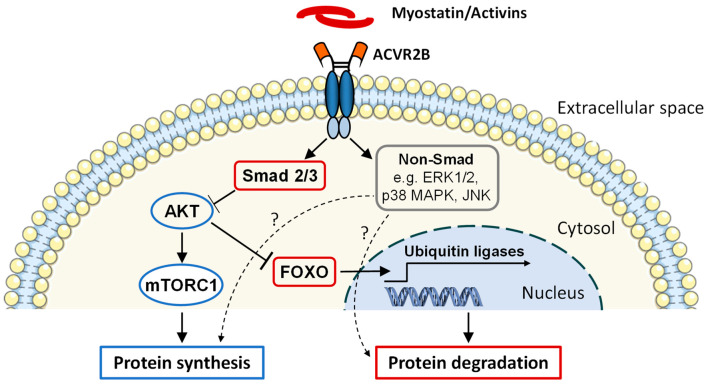
A simplified illustration of intracellular signaling induced by binding of myostatin or activins to their receptor activin receptor type 2B (ACVR2B). Cell membrane and DNA images were adapted from https://smart.servier.com (accessed on 23 February 2021) (Modified from Reference [135]).

Myostatin, GDF11 and activin A and B exert their effects through binding to activin receptors [123,135] expressed in many human tissues, including skeletal muscle [136]. Two types of activin receptors have been identified, and based on their molecular weight, named type I (low molecular weight) and type II (high molecular weight) receptors [127]. The ligand first binds to the activin receptor type II (ACVR2), which is a transmembrane protein consisting of an extracellular ligand-binding domain and an intracellular serine/threonine kinase domain [127,135]. It was first characterized by Mathews and Vale in 1991 [137], and two forms, i.e., ACVR2A and ACVR2B, have been identified [127]. The binding of the ligand to ACVR2A or ACVR2B enables the interaction of the ligand with type I receptor, i.e., activin receptor-like kinases (ALKs), further enabling the recruitment and phosphorylation of ALK by the activated kinase domain of ACVR2 [127,135]. This renders ALK active and results in phosphorylation of its downstream targets, such as the Smad2 and Smad3 transcription factors [127,135]. Smad2/3 then form a heterodimer complex that incorporates with Smad4 and translocates to the nucleus, where it influences the transcription of target genes [127,135]. Ultimately, the activation of Smad signaling results in inhibition of protein synthesis via mechanistic target of rapamycin (mTOR) [135,138] and may also promote protein degradation via enhanced nuclear translocation of forkhead box O3 (FoxO), followed by increased expression of ubiquitin ligases [135] (Figure 2), though the evidence for the latter is being debated [138]. In addition to the so-called canonical Smad signaling, other non-canonical pathways may also be regulated by ACVR signaling [139,140,141] (Figure 2). These Smad-independent pathways include p38 mitogen-activated protein kinase (p38 MAPK), extracellular signal-regulated kinase 1 and 2 (ERK1/2) and c-Jun NH_2_-terminal kinase (JNK), which have all shown increased phosphorylation by myostatin in muscle cells [139], a response that can have variable functions in muscle cells [127,135]. 

In addition, the complexity of Smad signaling in skeletal muscle was complicated by the discovery that several bone morphogenetic proteins (BMP) subfamily members activate Smad1/5/8, in parallel to the activin-Smad2/3 axis, thus promoting muscle hypertrophy. Smad2/3 and Smad1/5/8 operate in opposition, and indeed, BMP signaling inhibition results in muscle atrophy, unleashing myostatin/activin signaling [142].

### 3.2. Levels of ACVR2 Ligands in Cancer Cachexia and in Cancer Treatment

Activin A and B (inhibin βA and inhibin βB, respectively) are expressed in many human cancer cell lines and particularly in those that display a high degree of malignancy [131], which altogether could explain their role in inducing cachexia [143,144,145,146,147] and, potentially, survival [143,144,145,146]. Among the most extensively employed preclinical murine cancer cachexia models, the Colon 26 (C26) adenocarcinomas were reported to have markedly higher gene expression levels of activin A and myostatin when compared to Lewis lung carcinoma (LLC) tumors. Notably, growth of the C26 tumors also resulted in more pronounced cachexia at a similar time point with the same number of injected cells [13].

In patients with colorectal and lung cancer, high circulating activin A levels were associated with cancer cachexia [144,147,148,149], along with an independent negative prognostic impact [144] and reduced chemotherapy response [148], although in some other cancers, decreased activin-signaling was also observed [150]. In addition, based on a study by Miyamoto et al., a polymorphism in the activin A gene (*INHBA*) was found to predict survival in refractory metastatic colorectal cancer patients treated with regorafenib [151]. Conversely, in those very same studies showing elevated activin A, circulating myostatin was found decreased [147,149], similar to myostatin gene expression in gastric cancer patients with minimal or no weight loss [152,153]. Interestingly, circulating myostatin is substantially lower in humans than in mice [154], perhaps contributing to the poor translation of blocking myostatin alone [19]. More studies are needed to fully understand the role of activin A in muscle atrophy, also keeping in mind that low muscle size, as calculated from computed tomography (CT) scans, was not found to correlate with activin A levels [148].

In contrast to human and experimental cancer, there is not much evidence of upregulated ACVR2 ligands following cancer treatments. For instance, in mice, doxorubicin treatment did not induce mRNA expression of myostatin, activin A, Gdf11 or their receptor in the skeletal muscle or in the heart [15]. Similarly, animals exposed to Folfiri showed slightly higher levels of circulating activin A, which, however, were not statistically different from the vehicle-treated mice [57]. Since blockade of basal ACVR2 signaling is sufficient to increase muscle mass, it is also likely to counteract muscle wasting, compensating the catabolic stimuli in situations where ACVR2 ligands are not markedly elevated [15,57]. A number of studies conducted in healthy subjects would seem to support this idea, even though, due to the minimal or negligible muscle effects reported in some cases, more investigative efforts are required [18].

### 3.3. Strategies to Block ACVR2 Signaling

Given their important role in the regulation of muscle size, ACVR2B ligands are attractive targets for development of therapies to counteract muscle wasting. Many different strategies to block myostatin, and to some extent also other ACVR2 ligands, have been developed and successfully used in animals, and some have also been tested in humans [18,155]. The strategies developed cover all the steps from inhibition of synthesis to blockade of intracellular downstream signaling [155]. For instance, the synthesis of myostatin may be inhibited by RNA interference using antisense oligonucleotides that induce exon skipping on myostatin RNA, or with small interfering RNAs (siRNA) or short hairpin RNAs (shRNA) [155].

After synthesis and secretion, blockade can be achieved by different methods, such as administration of mutated myostatin or activin A or B pro-peptides, which bind myostatin and activins, thus inhibiting their activity [155,156], by treatment with neutralizing anti-myostatin or activin antibodies/peptibodies [157,158], or administration/expression of a native protein that binds myostatin and/or activins, thereby limiting their bioavailability. An example of such protein is follistatin, which is an endogenous inhibitor of myostatin, activins, GDF11 and some BMPs [133,135,155,159]. In addition, a very potent strategy to block myostatin and activins is the use of a soluble form of the extracellular domain of their endogenous receptor ACVR2B fused to Fc-region of IgG (sACVR2B-Fc, from here on referred to as sACVR2B) [160] or a neutralizing antibody against the activin receptor [11]. Both follistatin and sACVR2B sequester myostatin and activins, thus preventing their binding to the endogenous receptor [155].

Finally, the effects of myostatin and activins on target cells can be prevented by over-expression of dominant negative ACVR2B [117], or inhibition of ACVR2B or Smad2/3 synthesis [155]. In addition to these therapeutic strategies, different genetic models, such as constitutive, conditional and inducible knockout models, or heterozygous loss-of-function mutations, have been used to study the effects resulting from the lack of myostatin or activin A [155].

### 3.4. Effects of Blocking ACVR2 Signaling on Skeletal Muscle

Not surprisingly, multiple different strategies to block the function of myostatin or activins have been shown to increase muscle size and strength in healthy animals [134,155,161], as well as muscle size and, to some extent also function, in healthy humans, at least when multiple ligands were blocked [162,163]. Indeed, myostatin and activins synergize to regulate muscle mass, as the simultaneous blocking of these ligands was shown to drive even greater hypertrophy than the blocking of only one ligand at a time [134]. When blocked alone, myostatin inhibition appeared to induce the greatest increase in muscle mass and fiber size in mice, compared with other ACVR2 ligands [134]; however, whether the same also occurs in humans is unknown. Clearly, more clinical studies are needed to elucidate the relative physiological importance of different ACVR2 ligands.

Blockade of myostatin and activins in experimental models was found to prevent or attenuate muscle wasting associated with different diseases, including cancer, renal failure, heart failure, metabolic diseases, immobilization and sarcopenia, to name a few [164]. Improvement in muscle strength was also reported [164], although in this case, specific force might decrease because of larger increase in muscle mass relative to muscle force [165]. Direct targeting of myostatin by anti-myostatin antibodies was found to prevent the loss of muscle mass and function in mice bearing LLC tumors [158], whereas enhanced expression of follistatin by inhibitors of histone deacetylases, such as valproic acid or trichostatin-A, failed to improve cachexia in tumor-bearing rodents [166]. A causal role of blocking activin A was shown by systemically administering recombinant pro-peptide, which reversed activin A-induced cachectic wasting in mice [167]. Additionally, treatment with sACVR2B has been successful in the prevention of cancer-induced muscle wasting [9,10,13,35,98,168,169] and muscle weakness [10,168,169] in different pre-clinical models of cancer cachexia. This was shown to occur without an effect on physical activity or food intake [13,169]. In addition to blocking ACVR2 ligands, bimagrumab (BYM-338), a monoclonal antibody against ACVR2 receptors, was found effective in increasing muscle size and attenuating muscle loss in various animal and human studies [18], including in tumor hosts treated with chemotherapy [11]. More recently, in a phase 2 randomized clinical trial, ACVR2 blockade by bimagrumab led to loss of fat mass, gain in lean mass and metabolic improvements in type 2 diabetic patients who were overweight or obese [170], thereby supporting the use of such approach for the pharmacologic management of excess adiposity and metabolic disturbances. Keeping this in mind, the use of such approach in cancer patients, often characterized by extensive loss of adipose tissue mass, may be detrimental (see Section 3.6.2) [171]. It is also critical to understand that, although substantial muscle hypertrophy in theory enhances absolute muscle strength [172], improved body composition does not always translate into improved physical function [173]. As an example, recent bimagrumab treatment in older adults with sarcopenia who had six months of adequate nutrition and light exercise was reported to be safe and well-tolerated, increased lean body mass and decreased fat body mass, but did not improve physical function [174]. Lastly, a strategy to systemically block ACVR1 (ALK4/5) receptors by the inhibitor compound GW788388 was also shown to be effective in the preservation of body mass, muscle mass and muscle strength in murine cancer cachexia [175]. Altogether, these observations suggest that blocking ACVRs or their ligands by a variety of methods may represent potentially beneficial therapeutic strategies in cachexia.

Mechanistically, blocking ACVR2 signaling using sACVR2B was able to restore [35] or attenuate [169] decreased muscle protein synthesis and decreased mTOR colocalization with late-endosomes/lysosomes in C26 tumor-bearing mice [13], in line with the increased muscle protein synthesis observed after acute administration of sACVR2B in healthy wild-type mice [161]. In addition, sACVR2B prevented activation of the ubiquitin-proteasome system and induction of atrophy-specific ubiquitin ligases in muscles, and it increased satellite cell proliferation in C26-bearing mice [10]. However, the augmented ex vivo protein degradation in LLC tumor-bearing mice [169] and increased markers of activated ubiquitin proteasome system in C26-bearing mice [13] were not affected by sACVR2B [13,169]. Therefore, while the evidence of blocking ACVR2 signaling on increasing muscle protein synthesis is strong, the effects on muscle protein breakdown need further studies.

Of note, blockade of ACVR2 ligands was effective in preventing muscle mass loss also following administration of chemotherapy in mice. As an example, sACVR2B was able to counteract doxorubicin-induced losses of body and muscle mass, as well as muscle fiber size [14]. In a similar manner, treatment with sACVR2B completely preserved muscle mass and strength in animals administered with Folfiri [57]. Analogously, C2C12 myotubes exposed to Folfiri showed marked myofiber atrophy and elevated ERK1/2 phosphorylation, which were completely abolished by the combination with sACVR2B [17].

As a side note, one concern of blocking ACVR2 signaling in muscle was associated with negative changes in muscle oxidative metabolism in healthy and dystrophic mice [165,176]. Therefore, exercise as a co-treatment to prevent such changes has been suggested based on promising results in preclinical trials [176,177]. However, this effect of blocking ACVR2 signaling seems to be context-dependent, as in tumor-bearing mice, counteraction of myostatin [158] or ACVR2 ligands [35] was not shown to affect the muscle oxidative metabolism.

### 3.5. Blocking ACVR2 Ligands Improves Survival in Pre-Clinical Cancer Cachexia: Are the Effects Mediated by Skeletal Muscle?

Several studies showed that inhibition of ACVR2 signaling by systemically administered sACVR2B or antibodies against the receptor not only markedly improved muscle mass and function in cancer hosts, but also prolonged survival [10,11,12,13,146]. For instance, we [13] and others [10] showed that prevention of cancer-associated muscle wasting by sACVR2B resulted in marked improvement in survival without an effect on tumor growth in the C26 carcinoma model. Notably, survival was improved even when the treatment was not started until severe cachexia had already developed [10], thus supporting the use of such treatment to rescue overt cachexia. Similar blockade of myostatin and activins or genetic myostatin deficiency also prevented muscle wasting and improved survival in LLC and Apc^Min/+^ (multiple intestinal neoplasia of the murine Apc locus) models of cancer cachexia [105], as well as in inhibin-deficient mice [10,110], although in those cases, tumor growth was also partially inhibited, therefore likely playing a role in improving survival (see also Section 3.6).

The mechanisms underlying the positive pro-survival effects resulting from ACVR2 signaling blockade are still unknown. We investigated the potential mechanisms underlying the survival benefit with continued treatment with sACVR2B [13] with a pre-determined endpoint at the time point in which body mass change most strongly predicted survival (i.e., 11 days after tumor cell inoculation). In addition to unaffected tumor mass, treatment with sACVR2B did not influence tumor activin A mRNA expression, and increased Il-6 mRNA levels in the C26 tumors, thus suggesting that the prevention of cachexia, or the improved survival, are not mediated by modulating the expression of genes triggering cachexia in cancer cells [13]. At this time point, individual muscle weights were significantly higher in mice treated with sACVR2B compared with vehicle-treated tumor hosts. Thus, the preservation of muscle tissue may contribute to prolonged lifespan per se. This is supported by a large body of evidence showing that different strategies able to prevent muscle wasting in cancer cachexia result in improved survival [10,11,12,103,104,105,106,107,109,110,146,178,179]. It is possible that the preservation of some vital muscles, such as the major respiratory muscles, plays an important role in survival [100,101]. Indeed, diaphragm atrophy and weakness accompanied by ventilatory dysfunction have been previously reported in C26 tumor-bearers [13,24,99]. Importantly, ACVR2B signaling inhibition restored diaphragm mass [13], which may have contributed to the prolonged survival of these mice. However, more studies are required to confirm the importance of maintaining diaphragm mass and function during cancer cachexia.

It was recently shown that muscle-specific blockade of myostatin and activins by use of skeletal muscle-specific dominant negative ACVR2B-expressing transgenic mice did not improve survival with orthotopic pancreatic tumors originated from activin A high cell lines, despite the maintenance of body mass [146]. Similarly, also systemic ACVR2B blockade by sACVR2B, despite being effective in ameliorating cachexia, was not able to prolong survival in these hosts, whereas it prolonged life in mice implanted with pancreatic cancer cells expressing low activin A. The authors speculated that the lack of effects on survival in mice expressing muscle-specific dominant negative ACVR2B was possibly due to a critical role of activin signaling in other tissues, which were not directly targeted by the muscle-specific intervention. Another speculative hypothesis by the authors was related to the greater intrinsic propensity for cancer death in those mice, perhaps due to lower adiposity. However, the fact that the survival was not improved may not be entirely due to the absence of beneficial effects of ACVR2 signaling blocking in tissues other than skeletal muscle, especially since, as mentioned above, systemic blockade of these ligands did not ameliorate survival even in a tumor-model with high levels of activin A, unlike when pancreatic cancer cells expressing low levels of activin A were implanted [146]. Nevertheless, this study was critically important as it demonstrated that skeletal muscle-specific activin blockade alone may not be sufficient in a multi-systemic disease setting, such as cancer. Furthermore, the study strengthened the idea that mortality ultimately correlates with tumor activin A expression.

**Figure 3:**
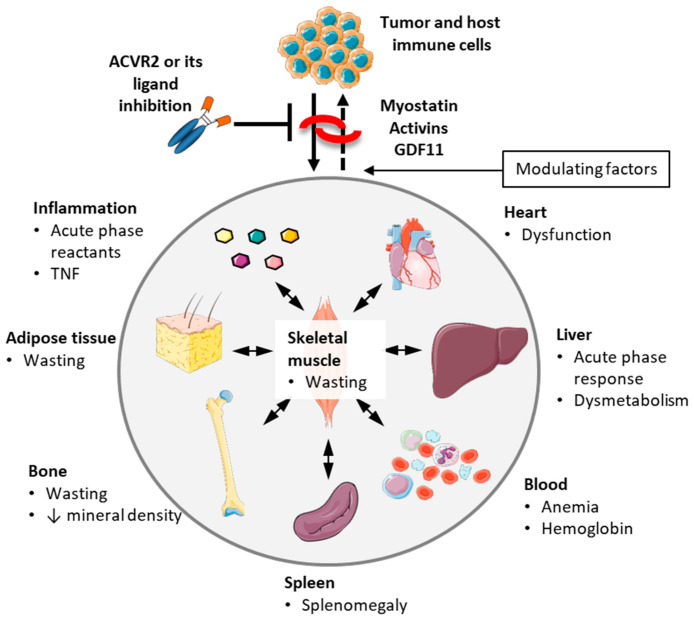
Simplified cartoon showing tumor-driven alterations potentially rescued by blocking ACVR2 or its ligands. Various factors modulate the treatment response, including tumor type, chemotherapy regimen, diet, physical activity, as well as specificity and timing of ACVR2 or its ligand inhibition. Some of the effects are stronger and more consistent than others and some organ effects may occur at least in part through rescuing muscle wasting. Organ images were obtained from https://smart.servier.com (accessed on 23 February 2021).

In addition to muscle wasting, alterations in other tissues and organs have been associated with onset of cachexia and worse prognosis in cancer [6,180]. For example, tumor growth was shown to induce increased levels of pro-inflammatory cytokines and acute phase response [35,181,182], increased spleen mass and expansion of myeloid-derived suppressor cells (MDSCs) [145,183], hematological changes, such as anemia and thrombocytosis [111], cardiac cachexia [184], fat depletion and adipose tissue browning [6,180], as well as alterations in gut microbiota [185,186,187] and bone abnormalities [188]. The relevance of each alteration to the development of cachexia and survival is poorly explored. It is thus possible that ACVR2 inhibition may prolong survival by targeting tissues other than the skeletal muscle, as discussed in the following section and summarized in Figure 3.

### 3.6. Non-Muscle Effects of Blocking ACVR2 Ligands

#### 3.6.1. Heart

In addition to skeletal muscle, atrophy of the heart, concurrent with altered cardiac function, has also been often observed in preclinical cancer cachexia [10,24,25,184]. In addition, cardiovascular complications are common, and may represent a major cause of death in cancer patients [111,184]. Blockade of ACVR2B signaling has shown beneficial effects on the heart as well [10,98,146,189], including reversing cardiac atrophy in both C26 mice and inhibin-deficient mice [10]. Similarly, blocking activin A also reversed activin A-induced cardiac wasting in tumor-bearing mice [167]. However, since not all studies have reported effects on the heart [12,13,158], further studies should be conducted to elucidate whether changes in cardiac mass and function are important and unappreciated features of cancer cachexia and whether ACVR2 ligand blocking has an impact on them.

#### 3.6.2. Adipose Tissue 

Skeletal muscle wasting is often accompanied by loss of adipose tissue in cachexia [6,7], and this is also true in preclinical models of cancer [10,13,98]. Interestingly, sACVR2B also preserved adipose tissue in animals bearing metastatic HCT116 [98], C26 [9,35] and LLC [9] tumors, as well as in mice exposed to Folfiri chemotherapy regimen [57]. This is in contrast with other studies using sACVR2B in C26 tumor-bearing mice in which loss of fat was not affected [10,13], and in doxorubicin-treated mice, fat loss was even exacerbated by sACVR2B [14]. Of note, although the effect of blocking ACVR2 ligands on fat mass is equivocal and context-dependent, a recent study shows that when loss of fat mass occurs, it happens as a counterbalance to increased muscle size, thus suggesting that ACVR2 blockade is not acting directly on the adipose tissue [190].

Fat loss was recently shown to play an important role in promoting cachexia and increased mortality in pancreatic cancer patients [171] and might therefore contribute to the finding that ACVR2 blockade did not improve survival in high-activin tumor-bearing mice despite maintenance of body mass [146]. However, the association between fat wasting and survival has not been observed in all studies [191], and thus further studies are needed to validate whether improved survival with inhibition of ACVR2 signaling results, at least in part, from preservation of fat mass. Of note, sarcopenic obesity often presents poorer prognosis in cancer compared to sarcopenia alone, although the presence of many confounders does not easily allow firm conclusions about the role of adipose tissue in cancer [192]. Hence, at this time, we can only speculate on the real contribution of fat wasting to overall survival in cancer.

In addition, white adipose tissue browning, which has been suggested to happen in cancer cachexia and to contribute to the progression of cachexia [180], may also be increased in some situations by blocking ACVR2 ligands [193]. However, markers of fat browning were not observed to be increased in C26-bearing mice, nor did the blocking of ACVR2 ligands have any significant effect [194], thus implying that adipose tissue browning may not be a major factor influencing outcomes and survival in this model of cachexia. However, more studies are needed to investigate the relevance of adipose browning in different cachexia models and in cancer patients. Considering the potential exacerbation of fat browning upon ACVR2 blockade, this may negatively impact on whole body energy homeostasis and should be taken into account.

#### 3.6.3. Blood Cells and Anemia

Cachectic tumor-bearing mice [13,195,196,197] and many cancer patients (e.g., Reference [198]) frequently present with anemia. Counteraction of ACVR2B ligands was able to reverse anemia in C26 hosts independent of the administration protocol [13] and in inhibin-deficient mice [110], while Apc^Min/+^ mice presenting myostatin gene inactivation were also free from anemia [105]. In contrast, sACVR2B treatment in mice implanted with LLC tumors was able to only partially alleviate the strong decrease in hemoglobin without changes in the hematocrit [12], while doxorubicin chemotherapy-induced anemia was not at all alleviated by sACVR2B [14]. Even though anemia may be an independent prognostic factor in cancer patients [198,199], and the prevention of anemia can be beneficial in C26 tumor-bearing mice [196,197], it may not be a major factor contributing to increased survival by sACVR2B, as also a “prophylactic” protocol prevented anemia without directly affecting survival [13]. In addition, in the very same study, the platelet count was increased in all tumor-bearing groups [13], independent of blocking ACVR2 ligands. Therefore, thrombocytosis might not be the major factor determining improved survival time by sACVR2B.

#### 3.6.4. Inflammation, Splenomegaly, Acute Phase Response and Tumor/Metastasis

Systemic inflammation is one of the hallmarks of cachexia [6,145,181]. Consistently, increased levels of circulating pro-inflammatory cytokines, such as TNF-α, IL-6 and MCP-1, have been reported in cachectic tumor-bearing mice, as well as in cancer patients [200,201,202,203]. Interestingly, administration of sACVR2B failed to correct [10,13,98] or only marginally impacted [12] the levels of these cytokines, as shown in several experimental conditions, thus suggesting that modulation of ACVR2 signaling is not directly involved in the regulation of the cytokine response during tumor growth.

Even though the blockade of ACVR2 ligands did not exert major effects on the levels of pro-inflammatory cytokines, it did impact other markers of inflammation. For instance, ample evidence revealed that tumor-bearing mice present increased acute phase response (APR) in both liver and skeletal muscle [13,35,182], in line with increased liver protein synthesis in tumor-bearing mice [13,204] and in weight-losing cancer patients [205]. Interestingly, liver protein synthesis, along with the phosphorylation of Stat3, were attenuated by sACVR2B treatment without effects on liver mass [13]. Although APR has been associated with impaired survival in humans [181] and in mice, whether this represents the potential additional mechanism by which sACVR2B alleviates cachexia [13,35] remains far from being elucidated.

Interestingly, activin A was shown to act in a paracrine fashion to stimulate melanoma growth and metastasization [206], thus suggesting that ACVR2 ligand blockers may simultaneously help in rescuing both muscle mass and immune function, hence also preventing tumor progression and cachexia [150]. Indeed, considering that activin A exerts pro-tumorigenic functions by promoting immunosuppressive activities in macrophages and Treg cells [207], ACVR2 ligand blockade may contribute to avoid tumor immune-escape, explaining the reported impact of sACVR2B on tumor growth and metastatic spread, independently from the presence of cachexia. Consistently, in studies conducted in myostatin-deficient Apc^Min/+^ mice and in LLC hosts [12,105], as well as in inhibin-α-deficient mice with gonadal tumors [10,110] and in a murine model of pancreatic ductal adenocarcinoma [146], blockade of ACVR2B ligands or signaling also resulted in partial attenuation of tumor growth and/or metastasis formation, which, in turn, may contribute to alleviation of cachexia and prolonged survival. This is an important point to consider, as in some cases, it is possible that the positive effects of the treatment may result from the antitumor effect of ACVR2 blockade rather than the preservation of skeletal muscle tissue. On the other hand, as also previously mentioned, the beneficial effects of ACVR2 ligand blockade did occur independent of effects on tumor mass [10,13] or tumor gene expression related to cachexia [13] in C26 hosts, thereby suggesting that the effect of blocking this pathway may be dependent on tumor type.

Considerable splenomegaly (i.e., increased spleen size) has also been observed in animals bearing tumors [13,22,23,35,208,209]. Along this line, a recent study showed that the splenomegaly induced by C26 tumors originated from erythrocyte engulfment and white blood cell proliferation [35]. Interestingly, this effect was significantly attenuated by starting administrations of sACVR2B before tumor inoculation [13], but not when the treatment was commenced after tumor implantation [35]. The former finding adds up to previous evidence showing that sACVR2B treatment alleviated splenomegaly in an animal model of β-thalassemia [210]. The expansion of the myeloid-derived suppressor cell (MDSC) pool has previously been associated with the development of cachexia and potentially also with survival [208]. Despite a marked effect on spleen size, the blockade of ACVR2 ligands did not consistently attenuate the mRNA expression of MDSC markers, that were found elevated in tumor-bearing mice [13]. Therefore, it may be argued that altered spleen size or MDCS expansion may not play a major role in survival in the presence of ACVR2 ligand blockade.

#### 3.6.5. Bone

Muscle and bone are known to interact in an endocrine-dependent manner [211]. According to this idea, muscle secretes factors (i.e., myokines) that can affect bone, whereas bone produces and serves as a storehouse for mediators (i.e., osteokines) that can target muscle. Keeping in mind that bone parameters were found strongly correlated with changes in lean mass, as well as with end-measures of muscle mass and muscle fiber cross-sectional area in doxorubicin-treated mice [14], it is possible that altered bone mass occurs at least in part secondary to changes in skeletal muscle [212,213,214].

Regardless, whether deregulations of the so-called ‘muscle-bone crosstalk’ take place in cachexia remains partially unknown. Loss of bone mass concurrent to evidence of muscle wasting has been previously observed in both metastatic [62,215] and non-bone metastatic tumor models [25,26,98,188,216], as well as upon administration of anticancer agents [57,63,217]. Interestingly, increasing muscle and lean mass via blocking of ACVR2 ligands has also been shown to improve bone parameters that were impaired by cancer [98] or following chemotherapy treatments [14,57], thus further supporting the idea that muscle and bone are regulated in tandem in cachexia.

However, previous evidence has highlighted how blockade of ACVR2 ligands can improve bone quantity and/or quality in different animal models by acting directly on bone [190]. Furthermore, counteraction of activin receptor ligands, but not of myostatin alone, was found to benefit bone mass, despite similar increases in muscle mass with both treatments [218]. This is of particular interest, especially considering that the abnormal activation of the signaling pathway downstream of the activin receptors is now known to play a role in the regulation of muscle and bone interaction, and that several ACVR2 ligands, including activin A, activin B, myostatin and GDF-11, have been reported to exert effects on both muscle and bone homeostasis, thereby contributing to the development of overt cachexia [147,219]. Together, these studies show that blockade of activin ligands is an effective measure to counteract bone and muscle loss in preclinical models of cancer- and chemotherapy-induced cachexia.

#### 3.6.6. Negative Side Effects of Blocking Myostatin, Activins and GDF11 and Other ACVR2 Ligands

The lack of high specificity of ACVR2 ligand and receptor blockers raised concerns about potential off-target effects. For example, the disruption of endogenous BMP-9 and BMP-10 signaling highlighted the potential occurrence of unwanted vascular effects [220]. Indeed, the clinical development of a soluble ACVR2B receptor designed by Acceleron Pharma (ACE-031) was prematurely terminated due to adverse effects, including nosebleed, gum bleeding, telangiectasia and erythema [221]. The cause was attributed to the cross-inhibition of BMP9 and BMP10, ligands involved in endothelial cell function. Further, a recent report showed that sACVR2B administration negatively impacts on testis, producing long-term hypogonadism and infertility [222], although a decrease in testis size was not observed in tumor-bearing mice with activin A blocked using its pro-peptide [167]. In addition, decrease in serum follicle stimulating hormone (FSH) was observed in humans after administration with both ACE-031 and ACE-011, a soluble ACVR2A-Fc [18]. ACE-011 administration also resulted in increased red blood cell numbers, possibly through inhibition of GDF11 [18], the latter also representing a positive response in cancer, as discussed above (see Section 3.6.3.). Nevertheless, because of such side effects, novel tools to block ACVR2 ligands and their signaling were recently generated. As an example, a soluble ACVR2B receptor modified to minimize vascular side effects (ACE-2494) was created and validated to be effective in murine models [223], although later discontinued in humans due to inconsistent profile of anti-drug antibodies [18]. In addition, the ACVR2 receptor antagonist bimagrumab (BYM-338), despite being effective in preventing muscle loss in murine cancer cachexia [11], promoted increased muscle size in some, but not all human trials [18]. The reader is referred to a recent comprehensive review by Suh and Lee to gain insights on the effects of new inhibitors of myostatin/activin signaling in clinical trials [18].

Altogether, the findings reported above emphasize the importance of stronger target specificity when developing future ACVR2 signaling inhibitors, especially for those in which long-term treatment is planned.

#### 3.6.7. Effects of Blocking ACVR2 Ligands in Cancer: Omics Approach

Systems biology is often necessary to understand the mechanisms of action of drugs that target several molecules in distinct tissues. In this regard, alterations in gut microbiota, previously shown to play a role in the development and progression of cancer cachexia, may potentially contribute to differences in survival time [185,186,187]. However, while altered gut microbiota in tumor-bearing mice was demonstrated, sACVR2B did not prevent the cancer-associated alterations in gut microbiota [224], suggesting that improved survival with ACVR2 ligand blocking is not mediated via changes in gut microbiome. As with the analysis of gut microbiota, the analysis of muscle and serum metabolomes, while providing new insight into metabolic alterations in cancer cachexia and potential new biomarkers for cachexia progression, did not provide any clear, plausible candidates to explain differences in survival [194]. However, ACVR2 blockade by sACVR2B was recently shown to rescue some of the metabolic alterations induced by chemotherapy, suggesting that ACVR2 ligand blockade could have beneficial effects on muscle and serum metabolomes in some cachectic conditions [16]. Moreover, administration of sACVR2B was found to be able to reverse or improve the dysregulated cardiac gene expression in cachectic mice bearing the HCT116 colorectal cancer, likely contributing to the improved cardiac function, despite that no effects on cardiac size were observed [98]. Lastly, a proteomics approach conducted in C26 tumor-bearing mice administered sACVR2B revealed improved oxidative phosphorylation (OXPHOS) proteome, which led to the identification of rescued nicotinamide adenine dinucleotide (NAD^+^) homeostasis [35]. These results open up new, interesting research questions and hypotheses for future studies aimed to elaborate on the mechanisms of cancer cachexia and improved survival.

## 4. Conclusions and Future Directions

A multitude of studies have demonstrated that administration of ACVR2 signaling blockers in pre-clinical cancer cachexia models leads to a number of positive health-related effects, including muscle growth or prevention of muscle wasting, maintenance of bone mass and bone mineral density, attenuation of hepatic protein synthesis, splenomegaly and anemia, in some instances, decreased tumor growth and metastases, and ultimately, improvement of survival. Given the importance of inter-tissue crosstalk in cachexia [180], it is possible that some of the above-mentioned beneficial effects take place secondarily to improvements of skeletal muscle. However, in many cases, it is impossible to separate the effects dependent on muscle size per se and those resulting from counteraction of the ACVR2B signaling that might be independent of changes in muscle mass. 

Based on findings by us and others, preservation of skeletal muscle tissue per se is critical for survival in cancer cachexia. In this regard, counteraction of muscle wasting by means of ACVR2 ligand blockers represents a promising strategy. However, further studies are needed to discriminate between the effects of maintenance of skeletal muscle (locomotor and respiratory) and the direct effects of, for example, systemic ACVR2 ligand blocking on non-muscle tissues. Strategies aiming to preserve individual muscles or muscle groups, such as the heart or the diaphragm, should be developed to the extent of assessing the importance of these vital muscles with respect to survival in cachexia, as induced by different tumor types. In addition, to validate whether maintenance of skeletal muscle is critically important to prolong life in cancer, investigations on the mechanisms associated with the preservation of muscle warrant further studies. For example, considering the multitude of organs, tissues and systems involved in cachexia, the interaction between muscle and other tissues in relation to survival requires supplementary investigations. Moreover, given the association between low muscle size/sarcopenia and poorer outcomes in human studies, it is of the utmost importance to generate observations in support of a lifestyle aiming at gaining and/or maintaining larger muscles. Future studies should also address the question of whether there is a causal link between the levels of cachexia-inducing factors, such as activin A, and survival, or whether these factors merely act as biomarkers of cachexia and disease progression.

The evidence from clinical studies showing that subjects with muscle wasting at time of cancer diagnosis also frequently present worse prognosis and shorter survival implies that having larger muscle mass to begin with might benefit patients’ outcomes in cachexia [5,27,69,83,84,85,86,87,88,89,90,91,92,225]. However, observations from our groups suggest that pharmacological enhancement of muscle mass prior to the cachectic stimulus is not sufficient to provide a survival benefit [13]. Hence, future studies are warranted to conclusively elucidate whether larger muscles at diagnosis may play an active role in improving cancer prognosis. Further, to the extent of validating exercise as a potentially powerful therapeutic strategy in cachexia, animal models that enable exercise interventions (e.g., resistance training [226,227]) should be developed [228,229] and tested in cancer [230] together with appropriate nutrition/nutraceutical strategies [231] to elucidate whether promoting better muscle mass and/or function also ultimately affects survival in cancer.

## Data Availability

No new data were created or analyzed in this study. Data sharing is not applicable to this article.
